# Genetic diversity and population structure of Chinese natural bermudagrass [*Cynodon dactylon* (L.) Pers.] germplasm based on SRAP markers

**DOI:** 10.1371/journal.pone.0177508

**Published:** 2017-05-11

**Authors:** Yiqi Zheng, Shaojun Xu, Jing Liu, Yan Zhao, Jianxiu Liu

**Affiliations:** 1 College of Forestry, Henan University of Science and Technology, Luoyang, Henan, P. R. of China; 2 Institute of Botany, Jiangsu Province and Chinese Academy of Sciences, Nanjing, Jiangsu, P. R. of China; National Cheng Kung University, TAIWAN

## Abstract

Bermudagrass [*Cynodon dactylon* (L.) Pers.], an important turfgrass used in public parks, home lawns, golf courses and sports fields, is widely distributed in China. In the present study, sequence-related amplified polymorphism (SRAP) markers were used to assess genetic diversity and population structure among 157 indigenous bermudagrass genotypes from 20 provinces in China. The application of 26 SRAP primer pairs produced 340 bands, of which 328 (96.58%) were polymorphic. The polymorphic information content (PIC) ranged from 0.36 to 0.49 with a mean of 0.44. Genetic distance coefficients among accessions ranged from 0.04 to 0.61, with an average of 0.32. The results of STRUCTURE analysis suggested that 157 bermudagrass accessions can be grouped into three subpopulations. Moreover, according to clustering based on the unweighted pair-group method of arithmetic averages (UPGMA), accessions were divided into three major clusters. The UPGMA dendrogram revealed that accessions from identical or adjacent areas were generally, but not entirely, clustered into the same cluster. Comparison of the UPGMA dendrogram and the Bayesian STRUCTURE analysis showed general agreement between the population subdivisions and the genetic relationships among accessions. Principal coordinate analysis (PCoA) with SRAP markers revealed a similar grouping of accessions to the UPGMA dendrogram and STRUCTUE analysis. Analysis of molecular variance (AMOVA) indicated that 18% of total molecular variance was attributed to diversity among subpopulations, while 82% of variance was associated with differences within subpopulations. Our study represents the most comprehensive investigation of the genetic diversity and population structure of bermudagrass in China to date, and provides valuable information for the germplasm collection, genetic improvement, and systematic utilization of bermudagrass.

## Introduction

The genus *Cynodon* (family Poaceae) contains 9 species and 10 varieties, with *Cynodon dactylon* (L.) Pers. (common bermudagrass) being the most widespread. Bermudagrass is found on all continents and islands between approximate latitudes of 45°N to 45°S [1]. The extensive use of bermudagrass in turf and pasture is due to its drought and heat tolerance and low maintenance requirements [2, 3].

Evaluation of genetic diversity and genetic relationships within germplasm can provide useful information for breeding programs [4]. The genetic diversity of bermudagrass has been screened and characterized based on morphology, isozyme electrophoretic patterns and DNA molecular markers. Previous studies indicated that the high degree of variation in morphological and reproductive characteristics and distributional patterns of bermudagrass [1, 2, 5–7]. Molecular markers have significant advantages over morphological and isozyme markers because they are uninfluenced by growth and environmental conditions and can be applied from any growth phase. A wide variety of molecular marker types have been applied to evaluate the genetic diversity of bermudagrass, including DNA amplification fingerprinting (DAF) [8–10], randomly amplified polymorphic DNA (RAPD) [11,12], amplified fragment length polymorphism (AFLP) [13–17], inter-simple sequence repeat (ISSR) [12, 18–21], simple sequence repeat (SSR) [21–23], peroxidase gene polymorphism (POGP) [12] and sequence-related amplified polymorphism (SRAP) [12, 24, 25] markers.

Bermudagrass is very abundant in China and widely distributed in tropical, subtropical and warm-temperate regions [26]. Several studies have investigated the genetic diversity of Chinese wild bermudagrass based on DNA molecular markers [14, 15, 17–19, 21–23, 25]. The studies mentioned above investigating the genetic diversity and relationships of bermudagrass accessions were mainly based on traditional cluster analysis which could provide an easy and effective method in estimating the genetic diversity of accessions [27]. Several other statistical methods including Bayesian cluster analysis, principal coordinate analysis (PCoA) and analysis of molecular variance (AMOVA) had been developed for analyzing the population structure, genetic diversity and differentiation of germplasm. Among these methods, Bayesian cluster analysis has been proven to be an efficient method to evaluate the population structure of germplasm collections, such as peanut [27], rice [28], apple [29], potato [30] and mung bean [31]. The Bayesian method applied in the STRUCTURE software [32] starts with a predefined number of genetic clusters, before running the algorithm, without any previous information about hypothesized genetic origin, sampling location [33]. Furthermore, estimating population structure is a crucial first step in association analysis as it could avoid false positives or spurious associations [32]. However, a comprehensive analysis of bermudagrass accessions genetic diversity and population structure in China is lacking.

Since the 90’s, the turfgrass research group at the Institute of Botany in Jiangsu Province & the Chinese Academy of Sciences have collected abundant wild bermudagrass germplasm mostly from China. But the genetic diversity and population structure of these germplasm have not been systematically studied by using molecular markers. Therefore, a comprehensive research on genetic diversity is still needed to evaluate these bermudagrass germplasm for its effective utilization in breeding. The present study was undertaken to systematically analyze genetic diversity and population structure in a set of 157 bermudagrass accessions using SRAP markers. The objectives of the study were: a) to assess levels of diversity present among accessions collected from different regions in China, b) to evaluate the population structure of these accessions.

## Materials and methods

### Plant materials and DNA isolation

No specific permissions were required for these locations. These materials were collected from roadside, river side, sea side, grassland, or open field, and were grown in 30-cm diameter pots in the greenhouse. Necessary fertilization and irrigation were made to ensure healthy and homogeneous materials. 157 natural bermudagrass accessions were analyzed collected from 20 provinces of China. Each accession listed in Table 1. Genomic DNA was extracted from leaves using the cetyltrimethylammonium bromide protocol [34]. DNA concentration was quantified using a UV spectrophotometer, and its integrity was verified by examining the fluorescence of ethidium bromide-stained samples on 0.8% agarose gels.

**Table 1:** Bermudagrass accessions analyzed in this study.

No.	Code	Origin	Latitude (N)	Longitude (E)	No.	Code	Origin	Latitude (N)	Longitude (E)
1	C007	Minhou, Fujian	26°05 ′	119°06 ′	81	C112	Sangzhi, Hunan	29°24 ′	110°06 ′
2	C011	Minhou, Fujian	26°05 ′	119°06 ′	82	C452	Yongzhou, Hunan	26°16 ′	111°37′
3	C019	Xiamen, Fujian	24°32 ′	118°10 ′	83	C474	Hengyang, Hunan	26°54 ′	112°36′
4	C021	Putian, Fujian	25°26 ′	119°00 ′	84	C468	Hengyang, Hunan	26°54 ′	112°36′
5	C163	Zhangzhou, Fujian	24°32 ′	117°32 ′	85	C477	Changsha, Hunan	28°12 ′	113°06′
6	C873	Minhou, Fujian	26°04 ′	119°13 ′	86	C480	Changsha, Hunan	28°12 ′	113°06′
7	C002	Yingtan, Jiangxi	28°15 ′	117°00 ′	87	C484	Changsha, Hunan	28°12 ′	113°06′
8	C003	Yingtan, Jiangxi	28°15 ′	117°00 ′	88	C496	Huaihua, Hunan	27°31 ′	110°03′
9	C114	Ji'an, Jiangxi	29°00 ′	114°45 ′	89	C504	Zhangjiajie, Hunan	29°16 ′	110°12′
10	C876	Shangrao, Jiangxi	28°36′	117°59 ′	90	C506	Zhangjiajie, Hunan	29°16 ′	111°00′
11	C034	Wuxi, Jiangsu	31°35 ′	120°12 ′	91	C507	Changsha, Hunan	30°35 ′	111°00′
12	C045	Xuzhou, Jiangsu	34°17 ′	117°10 ′	92	C508	Yichang, Hubei	30°35 ′	111°00′
13	C052	Xuzhou, Jiangsu	34°17 ′	117°10 ′	93	C732	Yichang, Hubei	28°08 ′	113°40 ′
14	C064	Yancheng, Jiangsu	33°23 ′	120°07′	94	C111	Yichang, Hubei	30°35 ′	111°00 ′
15	C065	Yancheng, Jiangsu	33°23 ′	120°07′	95	C519	Enshi, Hubei	30°15 ′	109°22′
16	C167	Nanjing, Jiangsu	32°03 ′	118°52 ′	96	C523	Enshi, Hubei	30°15 ′	109°22′
17	C736	Lianyungang, Jiangsu	34°36 ′	119°12′	97	C524	Enshi, Hubei	30°15 ′	109°22′
18	C815	Yancheng, Jiangsu	33°59 ′	120°23′	98	C526	Enshi, Hubei	30°15 ′	109°22′
19	C827	Rudong, Jiangsu	32°29 ′	121°10′	99	C528	Enshi, Hubei	30°15 ′	109°22′
20	C850	Sheyang, Jiangsu	33°59 ′	120°23′	100	C539	Qianjiang, Hubei	30°27 ′	112°48 ′
21	C858	Rudong, Jiangsu	32°29 ′	121°10′	101	C626	Shiyan, Hubei	32°52 ′	110°45 ′
22	C860	Rudong, Jiangsu	32°29 ′	121°10′	102	C627	Shiyan, Hubei	32°52 ′	110°45 ′
23	C028	Jinhua, Zhejiang	29°07 ′	119°32 ′	103	C632	Zhenping, Henan	33°04 ′	112°14 ′
24	C030	Hangzhou, Zhejiang	30°20 ′	120°12 ′	104	C634	Xinyang, Henan	33°04 ′	112°14 ′
25	C031	Fuyang, Zhejiang	30°20 ′	119°54 ′	105	C639	Xinyang, Henan	32°06 ′	114°07 ′
26	C737	Shaoxing, Zhejiang	30°00 ′	120°35′	106	C645	Xinyang, Henan	31°52 ′	114°08 ′
27	C870	Taizhou, Zhejiang	28°27 ′	121°31′	107	C650	Xinyang, Henan	32°09 ′	115°02 ′
28	C872	Wenling, Zhejiang	27°57′	120°51′	108	C681	Jiaxian, Henan	34°00 ′	113°12 ′
29	C038	Jiaozhou, Shandong	36°26 ′	120°00 ′	109	C690	Jiaxian, Henan	34°00 ′	113°12 ′
30	C039	Jiaozhou, Shandong	36°26 ′	120°00 ′	110	C701	Shangqiu, Henan	34°32 ′	115°38 ′
31	C040	Jiaozhou, Shandong	36°26 ′	120°00 ′	111	C705	Shangqiu, Henan	34°32 ′	115°38 ′
32	C164	Heze, Shandong	35°18 ′	115°15 ′	112	C719	Xinxiang, Henan	35°30 ′	113°51 ′
33	C708	Yantai, Shandong	37°30′	121°24 ′	113	C722	Xinxiang, Henan	35°30 ′	113°51 ′
34	C726	Zaozhuang, Shandong	34°52 ′	117°34 ′	114	C138	Kunming, Yunnan	25°07′	102°49′
35	C787	Tai'an, Shandong	36°12′	117°07′	115	C139	Kunming, Yunnan	22°30 ′	102°59 ′
36	C068	Hefei, Anhui	31°51′	117°13′	116	C560	Kunming, Yunnan	25°00′	102°42 ′
37	C078	Tunxi, Anhui	29°43′	118°20′	117	C574	Longling, Yunnan	24°38 ′	98°39 ′
38	C085	Chuxian, Anhui	32°18 ′	118°20 ′	118	C580	Dali, Yunnan	25°30 ′	100°12 ′
39	C100	Chuxian, Anhui	32°18 ′	118°20 ′	119	C596	Kunming, Yunnan	25°00 ′	102°42 ′
40	C101	Chuxian, Anhui	32°18 ′	118°20 ′	120	C597	Wuding, Yunnan	25°33 ′	102°10 ′
41	C141	Taiping, Anhui	29°40 ′	118°09 ′	121	C739	Kunming, Yunnan	25°07′	102°49′
42	C658	Jinzhai, Anhui	31°42 ′	115°51 ′	122	C711	Handan, Yunnan	36°34 ′	114°30 ′
43	C811	Hefei, Anhui	31°51′	117°13′	123	C713	Handan, Yunnan	36°34 ′	114°30 ′
44	C867	Dangshan, Anhui	34°26′	116°11 ′	124	C716	Handan, Yunnan	36°34 ′	114°30 ′
45	C182	Haikou, Hainan	20°02 ′	110°28 ′	125	C714	Handan, Yunnan	36°34 ′	114°30 ′
46	C185(1)	Sanya, Hainan	18°00 ′	108°54 ′	126	C832	Baoding, Yunnan	38°53 ′	114°26 ′
47	C188	Sanya, Hainan	18°00 ′	108°54 ′	127	C129	Xianyang, Shaanxi	34°25 ′	108°48 ′
48	C202(1)	Tongshi, Hainan	18°45 ′	109°31 ′	128	C133	Baoji, Shaanxi	34°27 ′	107°30 ′
49	C206(1)	Tongshi, Hainan	18°45 ′	109°31 ′	129	C134	Xianyang, Shaanxi	34°25 ′	108°48 ′
50	C207	Tongshi, Hainan	18°45 ′	109°31 ′	130	C587	Xingyi, Guizhou	24°43 ′	104°54 ′
51	C224	Baisha, Hainan	19°14 ′	109°28 ′	131	C588	Xingyi, Guizhou	24°43 ′	104°54 ′
52	C227	Baisha, Hainan	19°14 ′	109°28 ′	132	C592	Guiyang, Guizhou	26°36 ′	106°40 ′
53	C236	Danzhou, Hainan	19°30 ′	109°35 ′	133	C594	Guiyang, Guizhou	26°36 ′	106°40 ′
54	C242	Danzhou, Hainan	19°30 ′	109°35 ′	134	C812	Anshun, Guizhou	26°11′	105°54′
55	C254	Haikou, Hainan	20°02 ′	110°28 ′	135	C177	Xichang, Sichuan	27°57 ′	102°18 ′
56	C258	Haikou, Hainan	20°02 ′	110°28 ′	136	C180	Danba, Sichuan	30°53 ′	101°56 ′
57	C158	Shenzhen, Guangdong	22°15 ′	114°01 ′	137	C601	Xichang, Sichuan	27°53 ′	102°16 ′
58	C269	Zhanjiang, Guangdong	21°08 ′	110°31 ′	138	C604	Xichang, Sichuan	27°53 ′	102°16 ′
59	C269(1)	Zhanjiang, Guangdong	21°08 ′	110°31 ′	139	C611	Xinjin, Sichuan	30°14 ′	103°48 ′
60	C275	Zhanjiang, Guangdong	21°08 ′	110°31 ′	140	C612	Jiangjin, Sichuan	30°14 ′	103°48 ′
61	C280	Zhanjiang, Guangdong	21°08 ′	110°31 ′	141	C615	Chongqing	29°32 ′	106°33 ′
62	C311	Shenzhen, Guangdong	22°15 ′	114°06 ′	142	C616	Chongqing	29°32 ′	106°33 ′
63	C313	Shenzhen, Guangdong	22°15 ′	114°06 ′	143	C618	Chongqing	29°32 ′	106°33 ′
64	C380(1)	Yingde, Guangdong	24°06 ′	113°30 ′	144	C620	Chongqing	29°32 ′	106°33 ′
65	C360	Ruyuan, Guangdong	24°58 ′	113°15 ′	145	C801	Chongqing	29°32′	106°33′
66	C378	Yingde, Guangdong	24°06 ′	113°30 ′	146	C108	Urumqi, Xinjiang	43°56 ′	87°30 ′
67	C384	Yingde, Guangdong	24°06 ′	113°30 ′	147	C660	Urumqi, Xinjiang	43°56 ′	87°30 ′
68	C385	Yingde, Guangdong	24°06 ′	113°30 ′	148	C666	Kashi, Xinjiang	39°30 ′	76°00 ′
69	C803	Zhuhai, Guangdong	22°07′	112°45′	149	C670	Hetian, Xinjiang	36°54 ′	79°54 ′
70	C128	Guilin, Guangxi	25°18 ′	110°16 ′	150	C794	Kashi, Xinjiang	39°30′	76°00′
71	C391	Wuzhou, Guangxi	23°35 ′	111°12 ′	151	C672	Lanzhou, Gansu	36°00 ′	103°48 ′
72	C392	Wuzhou, Guangxi	23°35 ′	111°12 ′	152	C673	Lanzhou, Gansu	36°00 ′	103°48 ′
73	C406	Nanning, Guangxi	22°50 ′	108°12 ′	153	C675	Tianshui, Gansu	34°36 ′	105°48 ′
74	C413	Nanning, Guangxi	22°50 ′	108°12 ′	154	C676	Tianshui, Gansu	34°36 ′	105°48 ′
75	C415	Nanning, Guangxi	22°50 ′	108°12 ′	155	C824	Lingzhi, Tibet	29°36′	91°06′
76	C424	Baise, Guangxi	23°53 ′	106°25′	156	C825	Lingzhi, Tibet	29°36′	91°06′
77	C425	Baise, Guangxi	23°53 ′	106°25′	157	C826	Lingzhi, Tibet	29°36′	91°06′
78	C426	Baise, Guangxi	23°53 ′	106°25′					
79	C437	Liuzhou, Guangxi	24°18 ′	109°26′					
80	C450	Guilin, Guangxi	25°18 ′	110°18′					

### SRAP amplification

Twenty-six SRAP markers that produced high level of polymorphism and clear banding pattern were selected from the primers reported by Wang et al. [24] (Tables 2 and 3). SRAP amplifications for six samples (C007, C112, C634, C658, C807, and C826) were repeated twice to check for band repeatability. The amplifications from these samples repeatedly showed the same banding pattern. PCR amplifications were carried out in 20-μL reaction mixtures containing 2 μL of 1× buffer, 1.25 mM MgCl_2_, 0.26 mM dNTPs, 1 U *Taq* DNA polymerase, 0.2 μM primer and 50 ng DNA template. Amplifications were performed on a TC-412 thermal cycler (Techne, UK). PCR cycling conditions were according to Wang et al. [24]: an initial denaturation step of 94°C for 4 min, 35 cycles of 94°C for 1 min, 50°C for 1 min and 72°C for 10 s, with a final elongation step of 72°C for 7 min. PCR amplifications were repeated twice for each primer combination to ensure reproducibility. Amplified products were electrophoresed on 10% non-denaturing polyacrylamide gels [acrylamide-bis-acrylamide (19:1), 1× TBE] using DL1000 DNA marker (Tiangen Biotech, Beijing, China) as a molecular weight marker. Following electrophoresis, gels were stained with AgNO_3_ solution.

**Table 2:** Sequence-related amplified polymorphism (SRAP) primers used to detect polymorphisms.

Forward primer	Sequence (5’ to 3’)	Reverse primer	Sequence (5’ to 3’)
Me1	TGAGTCCAAACCGGATA	Em1	GACTGCGTACGAATTCAA
Me2	TGAGTCCAAACCGGAGC	Em2	GACTGCGTACGAATTCTG
Me3	TGAGTCCAAACCGGACC	Em3	GACTGCGTACGAATTGAC
Me4	TGAGTCCAAACCGGACA	Em4	GACTGCGTACGAATTTGA
Me5	TGAGTCCAAACCGGTGC	Em5	GACTGCGTACGAATTAAC
Me6	TGAGTCCAAACCGGAGA	Em7	GACTGCGTACGAATTGAG
		Em8	GACTGCGTACGAATTGCC
		Em9	GACTGCGTACGAATTTCA
		Em10	GACTGCGTACGAATTCAT

**Table 3:** Results of sequence-related amplified polymorphism (SRAP) marker amplification of the 157 bermudagrass accessions.

Primer combination	TNB^a^	NPB^b^	PPB^c^ (%)	PIC^d^
Me1-Em2	14	12	85.71	0.47
Me1-Em4	12	10	83.33	0.43
Me1-Em5	16	16	100.00	0.37
Me1-Em7	14	12	85.71	0.47
Me1-Em10	12	12	100.00	0.43
Me2-Em1	14	13	92.86	0.46
Me2-Em2	13	11	84.62	0.43
Me2-Em3	13	13	100.00	0.47
Me2-Em4	12	12	100.00	0.43
Me2-Em9	13	13	100.00	0.46
Me3-Em1	12	12	100.00	0.46
Me3-Em3	14	14	100.00	0.43
Me3-Em7	13	13	100.00	0.46
Me3-Em10	15	15	100.00	0.39
Me4-Em7	14	13	92.86	0.36
Me5-Em1	17	17	100.00	0.47
Me5-Em2	12	12	100.00	0.47
Me5-Em3	16	15	93.75	0.49
Me5-Em4	14	14	100.00	0.48
Me5-Em7	13	13	100.00	0.46
Me5-Em8	11	11	100.00	0.46
Me5-Em9	13	12	92.31	0.47
Me5-Em10	12	12	100.00	0.45
Me6-Em1	11	11	100.00	0.41
Me6-Em8	10	10	100.00	0.38
Me6-Em10	10	10	100.00	0.37
Average	13.08±1.74	12.62±1.77	96.58±5.72	0.44±0.04
Total	340	328		

^a^ Number of total bands

^b^ Number of polymorphic bands

^c^ Percentage of polymorphic bands

^d^ Polymorphism information content

### Statistical analysis

The distinct and reproducible bands of each SRAP marker were scored as either 1 (present) or 0 (absent). Genetic diversity parameters were calculated with the PIC (polymorphism information content). PIC for dominant markers was calculated as:

$$PIC=1-\left[f^{2}+{\left(1-f\right)}^{2}\right]$$

where *f* is the frequency of the marker in the data set. PIC for dominant markers is a maximum of 0.5 for *f* = 0.5 [35].

STRUCTURE software version 2.3.3 [32] which is a model-based Bayesian method was used to delineate the clusters of genetically similar accessions. The presumed number of subpopulations (*K*) was set from 1 to 15. For each run, the initial burn-in period was set to 100,000 with 100,000 Monte Carlo Markov Chain interactions. The number of subpopulations was determined using the Delta*K* method proposed by Evanno et al. [36]. Accessions were assigned to a subpopulation if the probability of membership was greater than 70% [37]. If membership was ≤70%, the accessions were assigned to the mixed subpopulation.

The NTSYS-pc version 2.1 software package [38] was used to calculate the genetic distance matrix. The unweighted pair-group method of arithmetic averages (UPGMA) [39] tree was constructed based on the genetic distance matrix generated by NTSYS-pc software using the Molecular Evolutionary Genetics Analysis (MEGA) 6.0 software [40]. A Mantel test [41, 42] was carried out to check goodness-of-fit between the similarity matrix and the cluster analysis results, as well as between geographic and genetic distances using the COPH (cophenetic values) option and MXCOP modules in NTSYS-pc.

Hierarchical analysis of molecular variance (AMOVA) was analyzed in GenAlEx 6.2 [43] to elucidate the extent of genetic variation among and within subgroups. Pairwise PhiPT value, an analogue of *F*_*ST*_ [44] to estimate of population genetic differentiation was also performed using GenAlEx with 999 permutations. Principal coordinates analysis (PCoA) was performed using GenAlEx based on genetic distance, and the first two principal coordinates were plotted in two-dimensional space.

## Results

### SRAP marker variation

Twenty-six SRAP markers yield clear, high-stability polymorphic bands. The total number of bands, the number of polymorphic bands, the percentage of polymorphic bands (PPB) and PIC were showed in Table 3. Amplification of the 26 SRAP markers across the 157 bermudagrass accessions generated 340 bands, of which 328 (96.58%) were polymorphic. The total number of bands scored per primer combination ranged from 10 (Me6-Em8 and Me6-Em10) to 17 (Me5-Em1), with an average of 13.08 bands per primer combination. Among these primers, Me1-Em4 generated the lowest percentage of polymorphic bands (83.33%); 18 primers (Me1-Em5, Me1-Em10, Me2-Em3, Me2-Em4, Me2-Em9, Me3-Em1, Me3-Em3, Me3-Em7, Me3-Em10, Me5-Em1, Me5-Em2, Me5-Em4, Me5-Em7, Me5-Em8, Me5-Em10, Me6-Em1, Me6-Em8 and Me6-Em10) yielded 100% polymorphic bands. PIC revealed the discriminatory power of the various SRAP markers. The mean PIC value for all markers was 0.44. The highest PIC values (0.49) was obtained for Me5-Em3 combination, followed by 0.48 for Me5-Em4, and 0.47 for Me1-Em2, Me1-Em7, Me2-Em3, Me5-Em1, Me5-Em2 and Me5-Em9. The primer combination Me4-Em7 had the lowest PIC value of 0.36.

### Population structure

**Fig 1:**
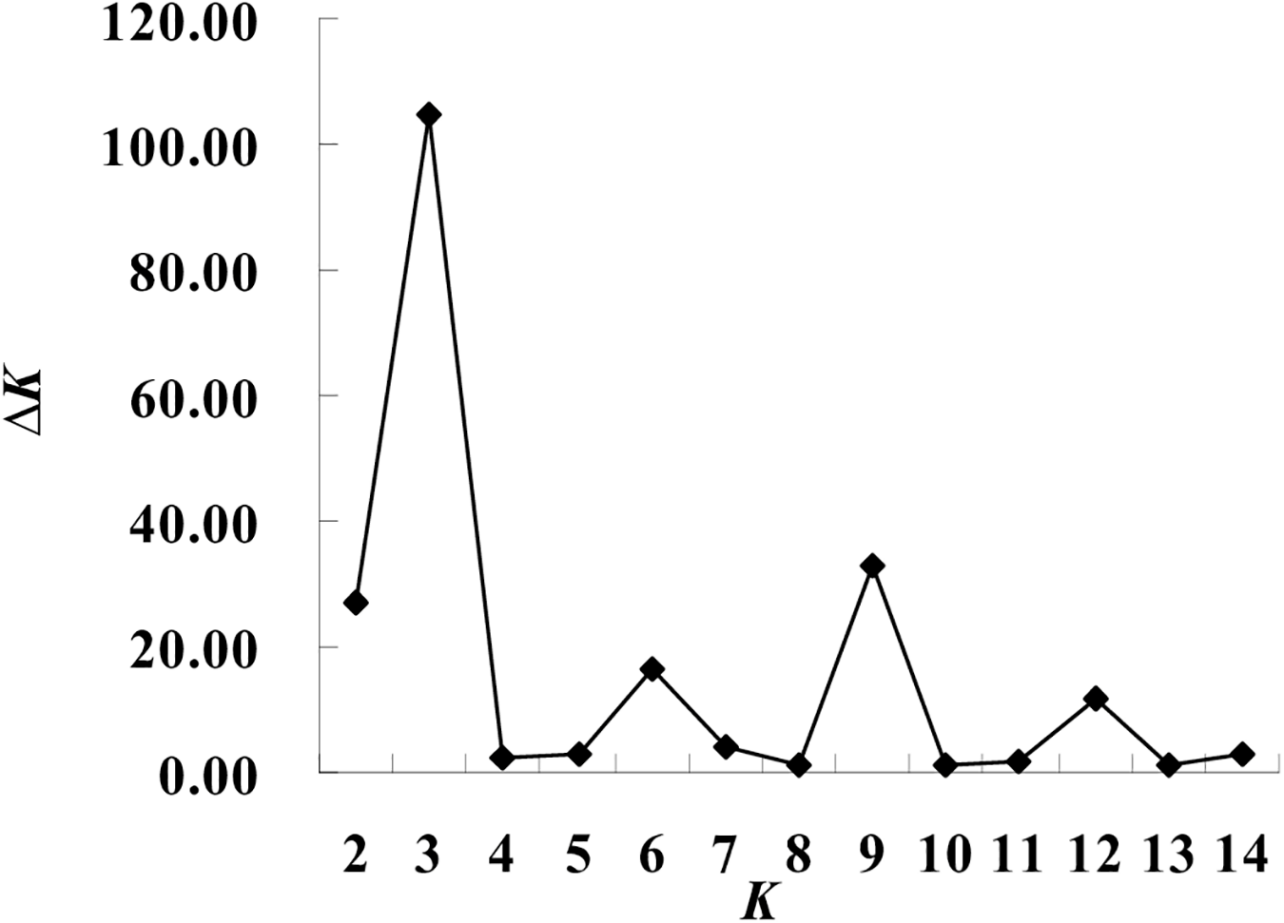
STRUCTURE estimation of the number of subgroups for the *K* values ranging from 1 to 15, by delta *K* (*ΔK*) values.

**Fig 2:**
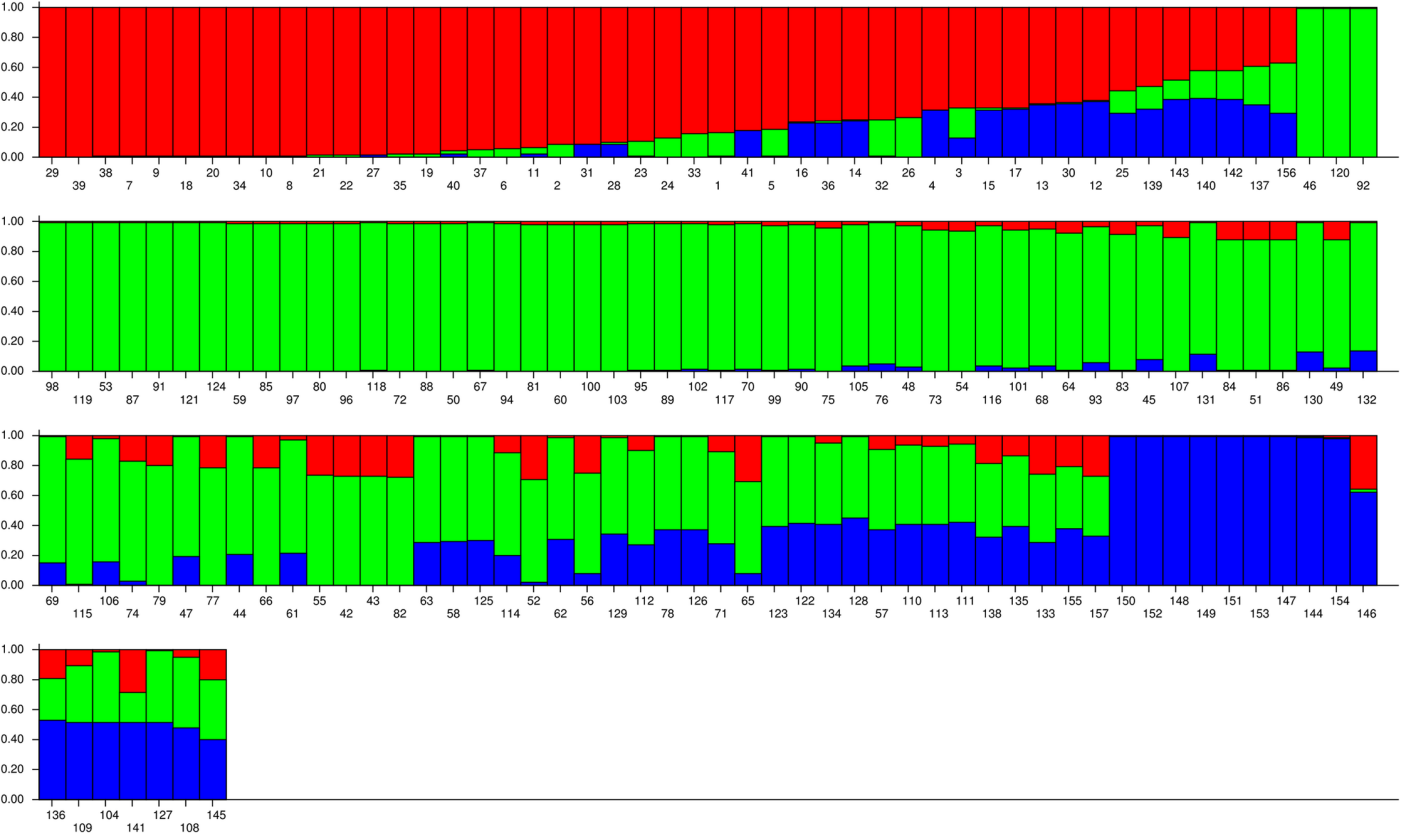
Population structure of 157 bermudagrass accessions based on sequence-related amplified polymorphism (SRAP) markers for *K* = 3. Each color represents one subgroup (subgroup C1 = red; C2 = green; C3 = blue) and the length of the colored segment shows the estimated membership proportion of each sample to designed group.

The population structure of the 157 bermudagrass accessions was analyzed by Bayesian based approach. Admixture model-based simulations were carried out by varying K from 1 to 15 with 5 interactions which showed the most suitable *ΔK* is 3, showed the most suitable number of subgroups to be three (Fig 1). In total, the 157 accessions can be grouped into three subpopulations (C1, C2 and C3). On the Basis of the membership fractions, the accessions with the probability of >70% were assigned to corresponding subgroups with others categorized as mixed subpopulation (Fig 2). In total, 33 accessions (21.02%) were assigned to subpopulation C1 from the eastern provinces including Fujian, Jiangxi, Zhejiang, Jiangsu, Anhui and Shandong. Subpopulation C2 consisted of 68 accessions (43.31%), 28 of which were collected from the southern provinces including Guangdong, Guangxi and Hainan, 26 of which were from central provinces including Hunan, Hubei and Henan, and 10 of which were from southwestern provinces including Yunnan and Guizhou, three of which from Anhui and one from Hebei province. Subpopulation C3 included nine accessions, mainly from northwestern provinces (four from Xinjiang and four from Gansu province) and one from Chongqing. The remaining 47 (29.94%) accessions appeared to have ancestry from more than one subpopulation, having *Q* values of less than 70% for both subpopulations. The mixed subpopulation contained 16 accessions from southwestern provinces consisting of Guizhou, Sichuan, Chongqing, Yunnan and Tibet, eight from eastern provinces consisting of Fujian, Jiangsu, Zhejiang and Shandong, eight from southern provinces including Hainan, Guangdong and Guangxi, four from northwestern provinces including Shaanxi and Xinjiang, seven from Henan and four from Hebei.

### Cluster analysis

**Fig 3:**
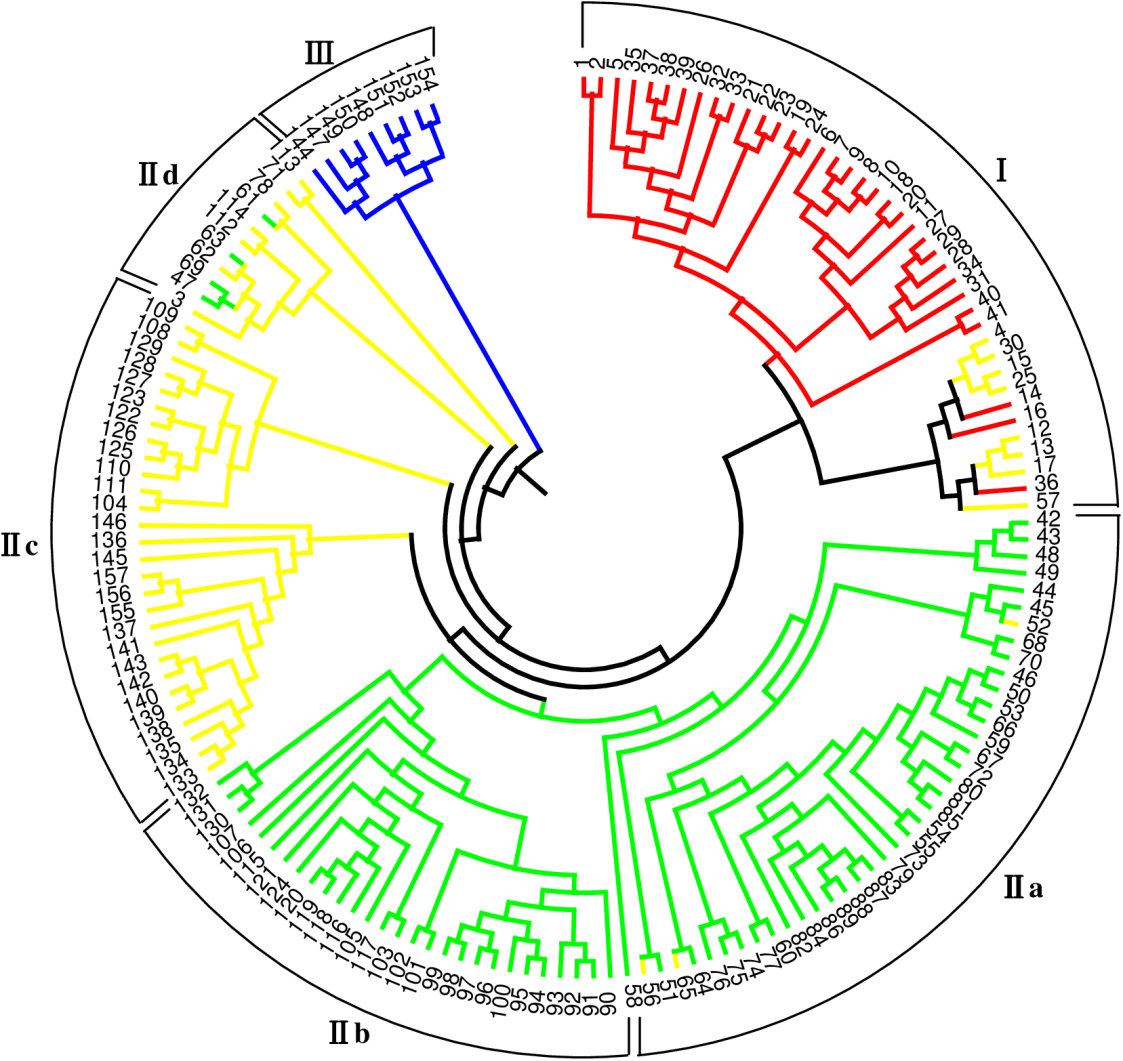
Unweighted pair-group method of arithmetic averages (UPGMA) dendrogram generated from SRAP data showing relationships of 157 bermudagrass accessions. Colors in the dendrogram correspond to population structure as identified in structure analysis. Each color represents one subgroup (subgroup C1 = red; C2 = green; C3 = blue, Mixed = yellow).

The genetic distance matrix ranged from a low of 0.04 between C596 and C597 (two accessions from Yunnan province) to a high of 0.61 between C003 collected from Jiangxi province and C794 collected from Xinjiang, with an average of 0.32. A dendrogram based on the genetic distance matrix of the SRAP data was generated using the UPGMA algorithm (Fig 3). In this dendrogram, the 157 bermudagrass accessions were clustered at a genetic distance of 0.344 into three clusters (Cluster I, Cluster II and Cluster III). The clustering results on the basis of genetic distance were generally consistent with the results from STRUCTURE analysis. Cluster I contained 41 bermudagrass accessions: five from Fujian, four from Jiangxi, 12 from Jiangsu, six from Zhejiang, seven from Shandong, six from Anhui and one from Guangdong province mainly from eastern China. This group consisted of all accessions from subpopulation C1 and eight accessions from mixed subpopulation. Cluster II contained 107 accessions and was further divided into four subgroups (IIa, IIb, IIc and IId). Subgroup IIa included 40 accessions: three from Anhui, 11 from Hainan, eight from Guangdong, nine from Guangxi and nine from Hunan. Subgroup IIb comprised 28 accessions: 17 from central China (four from Henan, nine from Hubei and four from Hunan), 10 originating from southwestern provinces (three from Guizhou and seven from Yunnan) and one from Hebei. Subgroup IIc included 28 accessions mainly from southwestern provinces (two from Guizhou, six from Sichuan, four from Chongqing and three from Tibet), five from Henan, four from Hebei, three from Shaanxi and one from Xinjiang. Subgroup IId contained 11 accessions: one from Fujian, one from Hainan, four from Guangdong, two from Guangxi, two from Henan and one from Yunnan. This group consisted of all accessions from subpopulation C2 and 39 accessions from mixed subpopulation. Cluster III contained nine accessions and was identical to subpopulation C3. These accessions were mostly from northwestern provinces (four from Xinjiang and four from Gansu) and one from Chongqing. In this dendrogram, accessions from identical or neighboring areas were generally, but not entirely, clustered into the same group or subgroup. Nevertheless, no significant correlation was found between geographic distance and genetic distance (*r* = 0.1657, *p* = 0.9986) based on the Mantel test.

### Principal coordinates analysis (PCoA) and analysis of molecular variance (AMOVA)

**Fig 4:**
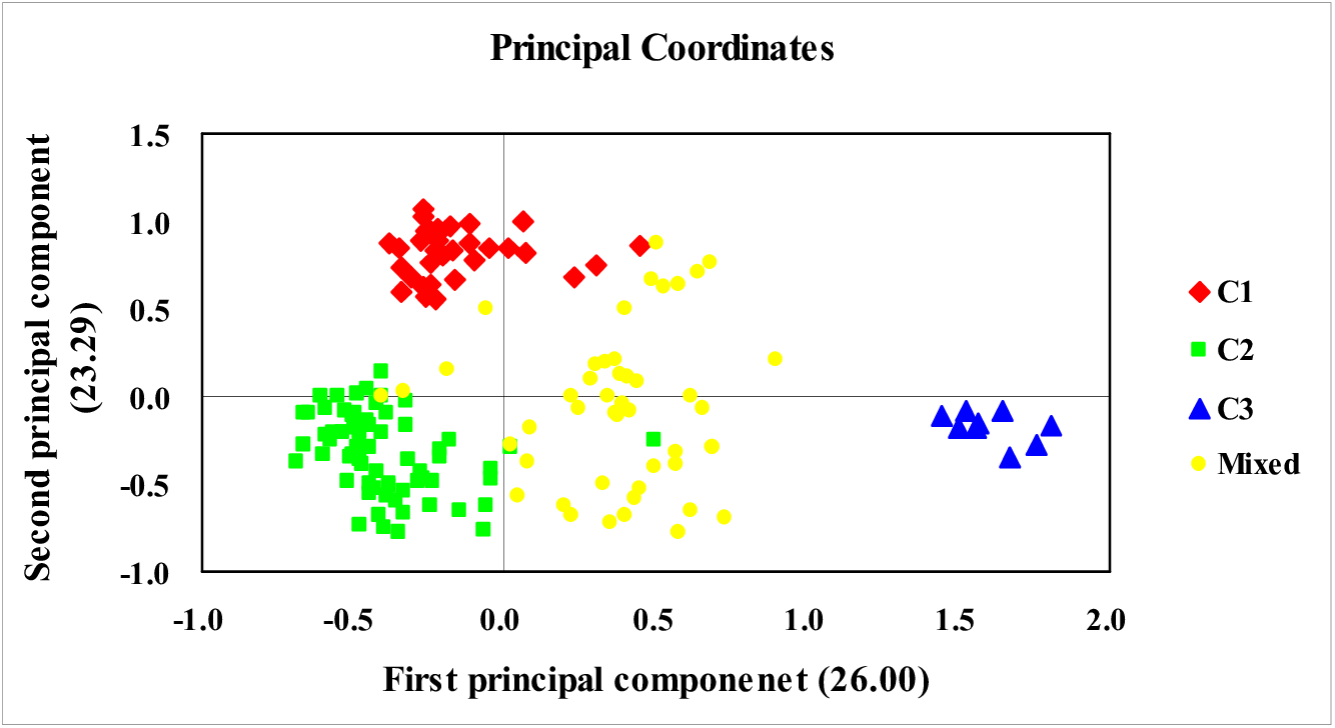
Scatter plot obtained from principal coordinate analysis of a genetic similarity matrix derived from 26 polymorphic sequence-related amplified polymorphism (SRAP) markers in 157 bermudagrass accessions.

Genetic relationships among bermudagrass accessions were further studied using Principal coordinate analysis. A two- dimensional scatter plot has shown that the first two PCoA axes accounted for 26.00% and 23.29% of the genetic variation, respectively (Fig 4). The PCoA plot revealed a similar grouping of accessions to the UPGMA dendrogram and STRUCTUE analysis. The subpopulations C1, C2 and C3 could be clear discriminated and the accessions from mixed subpopulation were placed in the middle of the three subpopulations.

An analysis of molecular variance (AMOVA) analysis was used to evaluate within and among subpopulation diversity components. Genetic differentiation among subpopulations was detected by AMOVA, the overall PhiPT values among subpopulations was 0.175 (*P*<0.001). The results of AMOVA indicated that majority of variance occurring within subpopulations accounted for 82% (*P*<0.001) of the total variation, and 18% (*P*<0.001) of variation was attributed to differences among subpopulations (Table 4). The pairwise PhiPT provided estimates of genetic distances between the subpopulations. The highest differentiation (0.416, *P*<0.001) was observed between subpopulation C1 and C3 and the lowest (0.093, *P*<0.001) was observed between Mixed and C2. Therefore, it could be inferred that C1and C3 subpopulations have diverged to a greater extent as compared to the mixed and C2 subpopulations (Table 5).

**Table 4:** Analysis of molecular variance (AMOVA) for the subpopulations as identified in STRUCTURE analysis.

Source of variation	d.f.	Sum of squares deviations	Estimaties of variance components	Percentage of variation (%)	P value
Among subpopulations	3	1060.514	8.827	18	0
Within subpopulations	153	6354.340	41.532	82	0
Total	156	7414.854	50.359	100	

**Table 5:** Pairwise estimates of PhiPT values among the subpopulations as identified in STRUCTURE analysis.

Subpopulation	C1	C2	C3	Mixed
C1	0.000			
C2	0.184	0.000		
C3	0.416	0.404	0.000	
Mixed	0.133	0.093	0.216	0.000

## Discussion

SRAP is a simple and efficient marker technique that has proven more informative for detecting genetic diversity than other DNA molecular marker systems [45]. In this study, SRAP markers were used to evaluate the genetic diversity of wild bermudagrass from China. Using 26 SRAP markers, 340 scorable fragments were obtained with an average of 13.08 fragments per marker which is higher than 5.4 fragments per marker detected by Gulsen et al. [12] in 182 bermudagrass accessions, and 9.0 fragments per marker reported by Wang et al. [24] in 24 bermudagrass cultivars, but is lower than 32 fragments per marker detected by Huang et al. [25] in 430 bermudagrass accessions. This showed that these SRAP markers are highly useful and can effectively be used in the genetic diversity studies. Out of these 340 fragments, 328 (96.58%) were recognized as polymorphic fragments which is higher than 91% reported by Wang et al. [24] and lower than 100% detected by Gulsen et al. [12] and Huang et al. [25]. The high level of polymorphism indicates that the high level of genetic diversity exists in the germplasm of bermudagrass. The level of polymorphism, however, generally was related to the number of accessions and their geographic origin, with a greater level of polymorphism among more accessions from wider geographic range compared to narrower range. PIC is a measure of allele frequencies at single loci or summed multiple loci. For dominant markers, the PIC values range from 0 to 0.5, where 0 indicates fixation of one allele and 0.5 means equal frequencies of alleles [35]. In the present study, the PIC value for SRAP markers ranged from 0.36 to 0.49 with an average of 0.44, also indicating that Chinese wild bermudagrass accessions displayed a wide range of genetic diversity, and these SRAP primers could develop abundant polymorphism which could be used to show differences between the samples analyzed in this study. The high genetic diversity in Chinese wild bermudagrass accessions may relate to biological characteristics and the geographic range of this species. Bermudagrass is a perennial, outcrossing, self-incompatibility [46, 47], and widespread grass species which may be one cause of the high genetic diversity. In addition, bermudagrass could clonal propagation by rhizome and stolon. Clonal and sexual propagation could result in many generations coexisting in a population. Such populations are insusceptible to genetic drift and are helpful to maintain genetic diversity [48, 49].

The results of the three analyses performed (UPGMA cluster, PCoA, and model-based method) agreed with the existence of three clusters or subpopulations. Despite minor differences, the results were largely consistent. Bayesian cluster analysis was used to infer the genetic structure and presence of possible populations and to estimate the ancestry of the sampled individuals [33]. In the present study, the model-based population structure analysis grouped the bermudagrass accessions into three ancestral groups: C1 group from East China, C2 group from South, Central and South West China, and C3 group from North West China. The accessions from North West China were separated from other accessions. The clear separation confirmed that accessions from North West China were genetically distinct from other accessions. This separation was also described by Xie et al. [23], in which 116 wild Chinese bermudagrass accessions from 14 provinces were grouped into two groups based on model-based population structure analysis, one group from North West China (Xinjiang province), and another from East, Central, South and South West China. The number of groups based on model-based population structure analysis was different between the present study and the study conducted by Xie et al. [23] which probably due to more accessions from East China examined in the present study. Admixture was also observed among few accessions with a proportion membership value of ≤70% in both the subpopulations. Most individuals belonged predominantly to one of the three subpopulations, while 47 (29.94%) were admixed according to the inferred subpopulation. Accessions from Jiangxi, Anhui, Hunan, Hubei and Gansu provinces had the lowest level of admixture (0.00%) and were homogenous, most likely because these accessions were subjected to limited exchange or diffusion. Accessions from Shaanxi, Sichuan and Tibet had the highest level of admixture (100.00%) and presented more admixtures, probably mixed ancestry from parents belonging to different gene pools. Admixture had been reported and is considered to the result of exchange of plant material between the areas and/or hybridization [50, 51]. As mentioned above, bermudagrass was outcrossing species and cross-pollination could result in admixture of alleles from adjacent regions. Meanwhile, exchanging of plant material between areas by animals and human activities may also form admixture.

The dendrogram constructed using the UPGMA clustering algorithm grouped the accessions into three clusters which was largely in accord with the result of the model-based method. All accessions of subpopulations C1, C2 and C3 were found in cluster I, II and III respectively, as identified in the distance-based method. These clusters, in most instances, revealed the majority of accessions that were geographically close were generally clustered into the same cluster except several accessions. For example, C019 collected from Fujian province was not clustered into the same cluster as other accessions from Fujian. The Mantel test also revealed little correlation between genetic and geographic distances. Similar results were obtained earlier in bermudagrass using AFLP markers [15] and SRAP markers [24] and may be due to outcrossing, self-incompatibility, or artificial transfer of accessions from one region to another.

The accessions of mixed subpopulation were clustered into two clusters (cluster I and cluster II) in UPGMA. Furthermore, most of the accessions in mixed subpopulation were located between clusters in the UPGMA tree as observed in the studies by Tyagi et al. [52] on the US Upland cotton. Eight accessions (C021, C039, C065, C031, C045, C052, C736 and C158) from mixed subpopulation were clustered into cluster I and located between cluster I and cluster II, 35 accessions were clustered into cluster II and located between cluster II and cluster III (Fig 3). Compared with the result conducted by the structure analysis, the accessions in mixed group were not identified in UPGMA tree. Thus, Bayesian cluster analysis can not only assign each individual to a hypothetical ancestral cluster(s) without any priori information [32], but also reveal the admixture that were not obvious using distance-based clustering methods.

AMOVA results obtained in this study indicate that there is a higher amount of genetic diversity within subpopulations than among subpopulation, indicating the existence of low genetic differentiation among subpopulations. A similar result was shown by Ling et al. [22] that 29.93% of the genetic variance existed among, while 70.07% within, the bermudagrass groups from Southwest China. This is a common situation that out-crossing and vegetative propagated perennial species are generally highly heterozygous and maintain high levels of genetic variation within populations [52–55]. Furthermore, the higher pairwise variation between C1 and C3, C2 and C3 could be explained from the fact that accessions from Northwest China being genetically differentiated from other accessions. The lower pairwise variation with C1 and C2, C1 and Mixed, C2 and Mixed may be due to collecting from adjacent regions and close kinship.

A principal coordinates analysis was conducted to further assess the population subdivisions identified using Structure. The PCoA analysis clearly separated the accessions into three gene pools which consistent with the results based on Structure, UPGMA, and AMOVA analysis. PCoA of collections from Northwest China showed that C3 subpopulation was very distinct, forming a separate group. In PCoA, accessions from Mixed subpopulation showed close association with other subpopulations demonstrated admixture in Structure analysis confirming their relatedness within the diverse gene pool.

In this study, we used simultaneously four methods including Bayesian clustering, UPGMA clustering, PCoA and AMOVA analysis to clarify the genetic relationship and genetic structure in the collection of 157 bermudagrass accessions. Despite minor differences, the results were largely consistent. By comprehensive analysis of the genetic diversity and population structure of Chinese wild bermudagrass germplasm, three ancestral gene pool were determined for the first time based on the different statistical methods. The UPGMA dendrogram revealed that accessions from identical or adjacent areas were generally, but not entirely, clustered into the same cluster. The results also provide evidence of abundant genetic diversity in these accessions and greater genetic variation within than among subpopulations. In summary, the results from the present study should lay foundation for further research, such as construction genetic linkage map, association studies, and molecular breeding studies.
